# Bacterial Biofilm and Titanium Implants: Mechanisms, Clinical Problems, and Surface Modification Strategies

**DOI:** 10.3390/ma19132919

**Published:** 2026-07-07

**Authors:** Julia Lisoń-Kubica

**Affiliations:** Department of Biomaterials and Medical Devices Engineering, Faculty of Biomedical Engineering, Silesian University of Technology, Roosevelta 40 Street, 41-800 Zabrze, Poland; julia.lison-kubica@polsl.pl

**Keywords:** biofilm, titanium alloy, biomaterials, surface engineering

## Abstract

Bacterial biofilms represent a major clinical challenge, being responsible for the majority of chronic infections and significantly reducing the effectiveness of antibiotic therapy. Their formation on implant surfaces, particularly those made of titanium and its alloys, is strongly associated not only with antimicrobial tolerance but also with persistent, hard-to-eradicate infections, implant loosening or failure, repeated surgical interventions, prolonged hospitalization, and increased morbidity. These complications contribute substantially to the growing problem of antimicrobial resistance and impose significant economic burdens on healthcare systems. This review discusses the mechanisms of biofilm formation, factors influencing bacterial adhesion, and the clinical implications associated with implant-related infections. Special attention is given to titanium-based biomaterials, including conventional Ti–6Al–4V and next-generation alloys such as Ti–13Nb–13Zr, highlighting their advantages and limitations in the context of biocompatibility and susceptibility to biofilm formation. Various strategies for combating biofilms are presented, including physical, chemical, and biological approaches, with emphasis on surface modification techniques. Advanced methods, particularly atomic layer deposition (ALD), are identified as promising solutions for creating uniform, antibacterial coatings, including those based on tin dioxide (SnO_2_). Such modifications offer potential for reducing bacterial adhesion, improving osseointegration, and enhancing long-term implant performance.

## 1. Introduction

Bacterial biofilms are currently recognized as one of the major contributors to chronic and persistent infections, representing a significant challenge in contemporary medicine. These structured microbial communities, embedded within a self-produced extracellular matrix, demonstrate a high level of resistance to both antimicrobial therapies and host immune defenses. Consequently, infections associated with biofilm formation are difficult to treat effectively and frequently require long-term therapy or surgical intervention. This issue is particularly relevant in the case of implantable medical devices, where microbial colonization of material surfaces may lead to serious complications, including implant failure and the necessity for revision procedures.

Titanium and its alloys are among the most widely used biomaterials in implantology due to their favorable mechanical properties, corrosion resistance, and excellent biocompatibility. They are commonly applied in orthopedic, dental, and cardiovascular systems. Despite these advantages, titanium-based implants are still susceptible to bacterial adhesion and subsequent biofilm formation. The implantation process itself creates a microenvironment that facilitates microbial colonization, while surface-related factors such as roughness, wettability, surface energy, and chemical composition play a crucial role in determining the extent of bacterial attachment. The limited effectiveness of conventional antimicrobial treatments, including antibiotics and antiseptics, has encouraged the development of alternative strategies aimed at preventing biofilm formation rather than solely treating established infections. In this context, surface modification of biomaterials has gained considerable attention as a promising approach to reduce bacterial adhesion and improve implant performance. Various physical, chemical, and biological methods have been proposed, including the use of functional coatings with antibacterial properties.

Advances in biomaterials engineering have led to the introduction of new-generation titanium alloys, such as Ti–13Nb–13Zr, designed to eliminate potentially harmful elements and enhance biocompatibility. Although these materials offer improved mechanical and biological characteristics, their resistance to microbial colonization remains insufficient. This has prompted further research into surface engineering techniques, particularly those involving antibacterial coatings based on metal ions, organic compounds, or metal oxides. Among these, tin dioxide (SnO_2_) has attracted increasing interest due to its antimicrobial activity combined with relatively low cytotoxicity and high chemical stability. When applied using atomic layer deposition (ALD), it is possible to obtain uniform, conformal coatings with precisely controlled thickness, even on complex and porous implant surfaces. Such modifications enable the tailoring of surface properties to simultaneously limit bacterial adhesion and support tissue integration. The aim of this review is to summarize current knowledge on biofilm formation on titanium implants, the associated clinical challenges, and available strategies for biofilm prevention and control. Particular emphasis is placed on modern surface modification techniques and antibacterial coatings, highlighting their potential to enhance the functionality and long-term reliability of implantable biomaterials.

A bacterial biofilm is a stable structure of microorganisms surrounded by extracellular polymeric substances (EPSs), forming a matrix composed of exopolysaccharides, nucleic acids, proteins, lipids, and other biological particles, firmly attached to both organic and inorganic surfaces. A biofilm may be formed by a single microbial species or may consist of a mixture of bacteria, fungi, archaea, protozoa, and yeasts. The structure of a biofilm is characterized by the irreversible adhesion of microbial cells to a substrate surface or to one another, as well as by the presence of channels that regulate the exchange of gases, nutrients, and antimicrobial substances. Extracellular polymeric substances (EPSs) promote the adhesion of microorganisms to both biotic and abiotic surfaces. Following attachment, continued EPS production leads to the formation of a matrix that surrounds and binds cells together, maintaining them in close proximity and enabling intercellular interactions within a confined space. The EPS matrix also provides mechanical stability and complex chemical microenvironments that are crucial for biofilm survival. As a result, microorganisms within the biofilm exhibit distinct phenotypes in terms of gene expression and growth rate, and the entire structure is characterized by increased resistance to antibiotics and to the action of the host immune system [1,2,3]. An essential element of biofilm function is intermicrobial communication, known as quorum sensing. This mechanism involves the production and detection of signaling molecules (autoinducers), the concentration of which increases with the number of cells. This enables the regulation of gene expression and the adaptation of phenotype to environmental conditions. Initially, it was believed that biofilms developed exclusively on solid surfaces, such as host tissues or implants. However, current studies indicate that they may also form in the absence of a solid surface [4]. Bacterial biofilms represent structures that are widely present in both natural and artificial environments, as well as in living organisms.

Bacterial biofilms are a major contributor to healthcare-associated infections (HAIs), accounting for a large proportion of persistent and recurrent clinical cases [5,6,7,8,9]. Their ability to adhere to both biological and abiotic surfaces and to form highly structured communities embedded in an extracellular matrix significantly enhances bacterial survival under hostile conditions [2,3,10,11]. This biofilm mode of growth is strongly associated with chronic infections and reduced susceptibility to antimicrobial agents, representing tolerance rather than classical genetic resistance [6,11,12]. From a clinical perspective, biofilm-associated infections are characterized by prolonged disease courses, frequent relapses, treatment failure, and increased risk of complications [9,13,14]. These factors translate into extended hospital stays, repeated surgical interventions, and substantial economic burden on healthcare systems [9]. Among clinically relevant biofilm-related conditions, implant-associated infections are particularly challenging due to the direct interaction between bacteria and biomaterial surfaces, which facilitates early adhesion and long-term persistence [9,15,16]. Despite numerous review articles addressing antimicrobial coatings and implant-associated biofilms [17,18,19,20,21,22], there is still a need for a focused and up-to-date synthesis that integrates recent developments in titanium-based biomaterials with advanced surface engineering strategies. In particular, emerging titanium alloys and novel deposition techniques such as atomic layer deposition (ALD) have not yet been comprehensively evaluated in terms of their combined impact on bacterial adhesion, biofilm formation, and osseointegration [23,24,25,26,27]. Therefore, this review aims to provide a structured and multidisciplinary overview of mechanisms of biofilm formation on implant surfaces, bacterial interactions with conventional and next-generation titanium alloys, and recent advances in surface modification strategies, with particular emphasis on ALD-based antibacterial coatings. By integrating perspectives from materials science, microbiology, and surface engineering, this work addresses a gap in the current literature and provides a consolidated framework for future development of anti-biofilm implant surfaces. Bacterial biofilms are a major contributor to healthcare-associated infections (HAIs), which are frequently characterized by chronicity, recurrence, and reduced treatment efficacy. These infections are associated with prolonged hospital stays, increased healthcare costs, and higher morbidity, particularly in patients with implanted medical devices [5,7,9]. From a healthcare system perspective, implant-associated infections impose a substantial economic burden due to prolonged hospitalization, repeated surgical procedures, and the need for long-term antimicrobial therapy [7,13,28].

This study presents a narrative review of the current state of knowledge regarding biofilm formation on implant materials, titanium alloys, and antibacterial surface modification strategies. The literature search was conducted using major scientific databases, including Scopus, Web of Science, and PubMed. The search strategy was based on combinations of the following keywords: “biofilm”, “titanium alloys”, “Ti–13Nb–13Zr”, “implant-associated infections”, “antibacterial coatings”, “biomaterials”, “surface modification”, and “osseointegration”. Boolean operators (AND, OR) were applied to refine the search results. The analysis primarily included publications from the period 2000–2025, with particular emphasis on studies published within the last decade to ensure up-to-date coverage of the topic. Earlier publications were included where they were considered foundational or highly relevant. The selection of literature was based on its relevance to the scope of this review, including mechanisms of biofilm formation, properties of titanium and its alloys, and approaches aimed at reducing bacterial adhesion and biofilm development on implant surfaces. Priority was given to peer-reviewed journal articles, original research papers, and review articles published in English. Due to the narrative nature of this review, a formal systematic selection protocol (e.g., PRISMA) was not applied. However, efforts were made to ensure a comprehensive and representative overview of the discussed topics.

## 2. Materials and Methods

Generative artificial intelligence (GenAI) tools were employed exclusively as supportive instruments for the development of schematic illustrations and graphical elements included in this manuscript. The tools used included Gemini and ChatGPT, which facilitated the transformation of complex biological and biomedical concepts into structured visual representations. No AI tools were used for data analysis, interpretation of results, or generation of scientific conclusions.

All AI-generated outputs were subjected to rigorous human oversight. The authors critically evaluated, substantially refined, and scientifically validated each generated figure to ensure accuracy, consistency with current knowledge, and alignment with the manuscript’s objectives. Final figures were manually edited where necessary to correct inaccuracies, improve clarity, and ensure compliance with scientific standards. The authors assume full responsibility for the integrity and correctness of all presented content.

To ensure transparency and reproducibility, the exact prompts used for figure generation are provided below.

Prompts using by GenAI to generate the figures:

All the figures were generated using Gemini, DALL-E 3.

## 3. Literature Search Strategy

A targeted literature search was conducted to identify relevant studies on bacterial biofilms, implant-associated infections, titanium-based biomaterials, and surface modification strategies, with particular emphasis on atomic layer deposition (ALD) coatings. The search was performed using major scientific databases, including Web of Science, Scopus, and PubMed. The following keywords and their combinations were used: “bacterial biofilm”, “implant-associated infection”, “titanium alloys”, “Ti–6Al–4V”, “Ti–13Nb–13Zr”, “antibacterial surfaces”, “osseointegration”, and “atomic layer deposition”. The search primarily covered publications from 2000 to 2026, with emphasis on recent studies from the last decade, while also including highly cited foundational works. Peer-reviewed original articles and review papers written in English were included if they were directly relevant to biofilm formation on biomaterials, implant-related infections, or antimicrobial surface engineering. Studies lacking relevance to biomedical implant applications were excluded. Additional relevant publications were identified through backward citation tracking of key articles.

## 4. Bacterial Biofilm Formation

### 4.1. Statistics of Bacterial Biofilm and Infections

In recent decades, bacterial biofilms have increasingly been recognized as a major factor in the pathogenesis of chronic infections, disrupting homeostasis [29]. Available statistical data indicate that [30] biofilms are responsible for 60–80% of all bacterial infections in humans. According to the U.S. National Institutes of Health (NIH) [5], the development of biofilms within the body is associated with more than 80% of microbial infections in humans, posing a serious threat, particularly in cases of inappropriate treatment. Similar findings have been reported for chronic and hard-to-heal wounds, in which the presence of biofilm has been identified in as many as 78% of cases. The scale of the problem is also enormous from an economic perspective. In 2019, approximately USD 260 billion was spent worldwide on the treatment of wounds associated with complications following biofilm formation [6]. Other sources confirm that biofilms are present in approximately 80% of chronic infections, significantly hindering effective therapy. Similar values (65–80%) are also reported by Wu et al. [31], who identify biofilms as the dominant mechanism underlying chronic infections. In turn, a review by Sharma et al. [6] demonstrates that up to 70% of all microbial infections are associated with the presence of biofilms, particularly in infections of bones, lungs, and the urinary tract. Biofilms represent a serious clinical problem due to the low efficacy of drugs and the high level of resistance to antimicrobial drugs. Studies show that bacterial biofilms not only cause infections but also exhibit resistance to antibiotic therapy. The lack of dedicated treatment methods further prolongs the therapeutic process [31]. The use of antibiotics and antiseptics does not constitute an effective method for creating a so-called barrier between the environment and the substrate surface, as biofilms require the application of significantly higher concentrations of antibiotics than those used to eliminate individual bacterial cells [10]. Moreover, the occurrence of biofilm-associated infections in the hospital environment is particularly concerning. Such infections account for more than 65% of all infections, of which as many as 80% are chronic, while approximately 60% involve bacterial infections in the human body. Available data clearly indicate that biofilms represent a major challenge both for effective infection control and for patient treatment. [28]. An analysis of the literature, as well as the number of queries and searches in the PubMed database, indicates an increase in the number of publications devoted to infections developing as a result of biofilm formation following implant placement. Over the past 40 years, a clear upward trend has been observed in this area. Moreover, due to growing awareness of the problem, over the past 20 years, the number of patents related to measures preventing biofilm formation has increased by nearly 80%, highlighting the significance of this issue.

### 4.2. Scheme of Biofilm Formation

**Figure 1:**
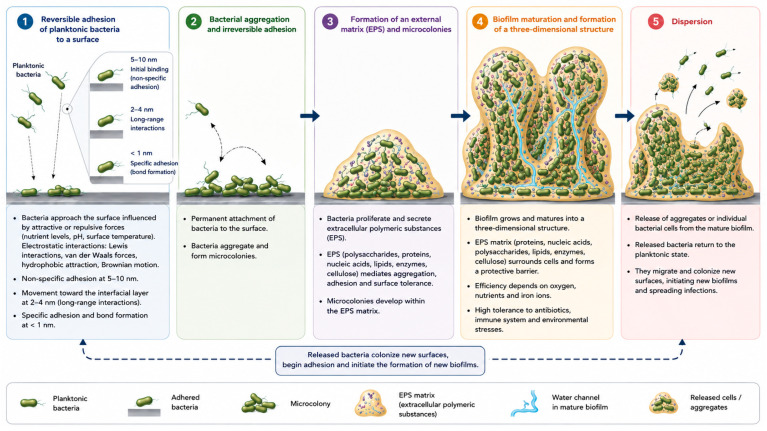
Biofilm formation cycle.

The process of biofilm formation constitutes the bacterial life cycle and includes the following stages [31,32,33] (Figure 1):

1. Reversible adhesion of individual planktonic bacteria to a surface, where bacterial adhesion is influenced by attractive or repulsive forces generated by nutrient levels, pH, and surface temperature. Individual bacteria are subject to electrostatic interactions, such as Lewis interactions, van der Waals forces, hydrophobic attraction, and Brownian motion. In the initial phase, when bacteria are located at a distance of 5–10 nm from the substrate surface, the initial stage of binding occurs, referred to as nonspecific adhesion. As bacteria approach the surface to a distance of 2–4 nm, long-range interactions facilitate their movement toward the interfacial layer. Ultimately, at distances below 1 nm, specific adhesion, associated with the formation of bonds between bacteria and the surface, becomes possible.
2. Bacterial aggregation and irreversible adhesion, that is, the permanent attachment of bacteria to the surface.
3. Formation of an external matrix composed of multilayered biomolecules, in which microcolonies and EPS secretion lead to the formation of the external matrix. The secretion of polysaccharides by biofilm-forming strains enables aggregation, adhesion, and surface tolerance, thereby facilitating surface colonization.
4. Biofilm maturation and the formation of a three-dimensional structure, based on self-produced components of the extracellular matrix. The maturation process initially involves the formation of the bacterial cell network through proliferation, intercellular adhesion, and the formation of an extracellular EPS matrix surrounding the microorganisms and constituting a barrier impeding the penetration of drugs and immune system cells. The composition of the EPS matrix includes, among others, proteins, nucleic acids, cellulose, enzymes, lipids, and polysaccharides. The efficiency of biofilm maturation depends on environmental factors such as the availability of oxygen and nutrients, as well as the presence of iron ions, which are essential for the proper growth of bacterial cells.
5. Dispersion is the release of aggregates or individual bacterial cells from a mature biofilm into the environment. Subsequently, the released bacteria regain a planktonic state and migrate in search of new surfaces for colonization. Through this process, biofilms expand, initiating infections at sites through renewed proliferation and the formation of new structures. Characteristic features of biofilms include high tolerance to unfavorable environmental conditions, such as temperature fluctuations, reduced humidity, exposure to antibiotics, and components of the immune system, including macrophages and antibodies. The heterogeneous structure of a biofilm promotes the growth and proliferation of microorganisms that become distinct from planktonic forms, leading to increased resistance to antibiotics and antiseptic drugs.

**Figure 2:**
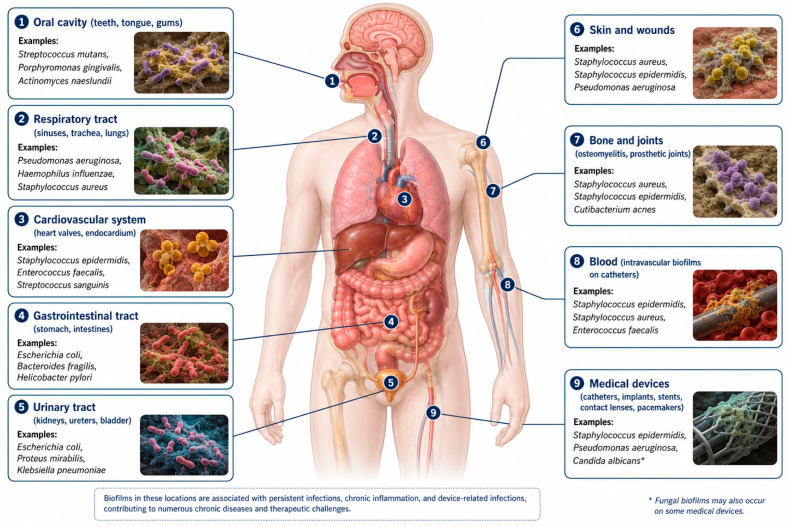
Presence of bacterial biofilms in various human tissues and organs.

Statistical data indicate that by 2050, bacterial infections may be responsible for up to 10 million deaths annually [10]. Biofilms are present in the human body, where they are associated with numerous chronic diseases and may disrupt homeostasis, that is, the internal balance of the organism. Biofilms represent a serious therapeutic challenge due to increased resistance and the ability to evade both diagnostics and immune responses. It has been demonstrated that biofilms are ubiquitous and form in various tissues of the body (Figure 2), as well as on biotic and abiotic surfaces, including medical devices.

### 4.3. Places of Biofilm Formation

Bacterial biofilms may form, among others, on wound surfaces; in the oral cavity (on teeth and gingiva); within the urinary, respiratory, and circulatory systems; and within bones. Particularly susceptible to colonization by microorganisms are the surfaces of foreign bodies, such as various types of implants, as their implantation into the body creates favorable conditions for bacterial adhesion and biofilm formation [8]. The body has a limited capacity for natural clearance, and bacteria rapidly colonize the surfaces of foreign bodies, leading to chronic infections that are difficult to eradicate [11,31,34,35,36]. In the case of implants such as joint prostheses, dental implants, artificial heart valves, and vascular and urinary catheters, the surrounding environment promotes the formation of bacterial biofilms. The surface of implants, particularly those made of polymers or low-roughness metals, facilitates bacterial adhesion. Following implantation, host proteins such as fibrinogen and fibronectin also adsorb onto the surface, which further facilitates bacterial adhesion. Conditions present in the vicinity of an implant, such as the limited flow of body fluids, local hypoxia (oxygen deficiency in a given area of the body), reduced immunoreactivity of the body (the ability to elicit an immune response), and the absence of mechanisms for mechanical bacterial clearance (present, for example, in the intestines), also promote bacterial growth, leading to the formation of a biofilm structure. Under such conditions, bacteria produce an extracellular matrix (EPS) that protects them from the effects of antibiotics and the immune system. As a result, microorganisms embedded in biofilms can survive antibiotic doses up to 1000 times higher than those tolerated by their planktonic counterparts [9]. Biofilm presence on implants often leads to chronic infections that may not produce acute symptoms but persist over prolonged periods, resulting in tissue degradation, pain, and even the need for implant removal [8,37]. Biofilms on implants represent not only a microbiological problem but also a serious material and clinical challenge. The high resistance and ability to survive make biofilms a frequent cause of postoperative complications, chronic infections, and a significant increase in healthcare costs [38].

To sum up, biofilm-forming bacteria pose a significant challenge to modern medicine due to their prevalence and their major role in the pathogenesis of chronic infections. The structure of the biofilm, based on the extracellular matrix (EPS), provides microorganisms with a range of adaptive benefits. From the bacteria’s perspective, the most important advantages of biofilms include increased resistance to antibiotics and antiseptics, protection against the host’s immune response, and the ability to communicate with one another (quorum sensing), which enables the regulation of gene expression and adaptation to environmental conditions. Biofilms also provide stable growth conditions and efficient utilization of available resources. Furthermore, in a clinical context, the presence of biofilms is associated with numerous disadvantages. Above all, it leads to the development of chronic infections that are difficult to eradicate and often require long-term treatment. Biofilms significantly reduce the effectiveness of standard antibiotic therapy, which can lead to recurrent infections, chronic inflammation, and tissue damage. Additionally, they generate high treatment costs and pose a significant problem in the hospital setting. Limitations related to the diagnosis and treatment of biofilms are also a key issue. These include, above all, the lack of effective, targeted treatment methods, limited drug penetration through the EPS matrix, and difficulties in detecting biofilms using standard diagnostic methods. Microorganisms in biofilms can exhibit antibiotic tolerance that is up to several hundred to a thousand times greater than that of planktonic forms, which significantly hinders effective treatment. Despite these challenges, knowledge about biofilms has broad clinical applications. It is crucial in the treatment of chronic wounds; infections associated with implants (such as joint prostheses, heart valves, or catheters); and infections of the urinary, respiratory, and oral tracts. Understanding the mechanisms of biofilm formation enables the development of new therapeutic strategies, including antimicrobial coatings for implants, targeted therapies against biofilms, and methods to prevent their formation. In summary, biofilm represents a complex and multifaceted clinical problem requiring further research into effective diagnostic and therapeutic methods, as well as the implementation of innovative solutions in medical practice.

### 4.4. Mechanisms of Bacterial Biofilm Formation and Development

Bacterial colonization of implant surfaces is associated with adhesion phenomena and attachment mechanisms, including physical force interactions, which lead to irreversible bacterial attachment to the surface and represent one of the main causes of biofilm formation [8,10,39]. Surface roughness plays a significant role, as increased roughness promotes the accumulation of bacteria within surface irregularities, whereas smooth surfaces with low roughness limit the adhesion process [40]. Even hybrid surfaces, used for example in dental implants, which combine partially smooth and rough regions, favor the accumulation of bacterial biofilms on the rougher areas. Moreover, surface wettability also plays a role. The more hydrophobic the surface becomes, the greater the bacterial adhesion [13]. Despite similar roughness values or surface characteristics, the chemical composition of the material itself also influences the formation of bacterial biofilms. For example, the Co–Cr–Mo alloy exhibits lower susceptibility to colonization than the more susceptible Ti–6Al–4V alloy [41]. In addition, surface energy governs protein adsorption and the initial stages of microbial attachment. Surfaces with higher charge and surface energy promote protein adsorption, followed by the adhesion of bacterial cells, which in turn precedes bacterial adhesion to the surface [42]. The intrinsic antimicrobial activity of metallic biomaterials is closely related to their ion release profiles and associated cytotoxic effects. In the case of Co–Cr–Mo alloys, the release of Co and Cr ions may contribute to reduced bacterial viability through ion-induced oxidative stress and membrane disruption; however, this effect is often accompanied by increased cytotoxicity concerns in host tissues [43,44]. In contrast, titanium- and aluminum-based alloys are characterized by excellent biocompatibility and chemical stability, but they generally lack significant intrinsic antibacterial activity, which may increase their susceptibility to bacterial adhesion and biofilm formation in the absence of surface modification [16,24,25]. Studies investigating pathogen adhesion involve comparisons concerning the effects of biomaterial types, with particular emphasis given to implant surface properties and their influence on biofilm formation [14,45,46,47]. In addition, there are biological factors that are closely associated with specific bacterial strains and proteins within particular body systems, resulting in a mechanism that enables stable attachment, especially under bodily fluid conditions [48]. Furthermore, environmental factors such as reduced patient immunity, tobacco smoking, the duration of the implantation procedure, and the organism’s microbiology may also promote biofilm formation. There is also the phenomenon known as the race for surface, i.e., the relationship between the rate of adhesion and bacterial growth in the form of a biofilm and the tissue integration of an implant [49]. In the case of increased aggregation, that is, clustering of bacteria, the risk of infection increases significantly. In such a case, implant removal followed by reimplantation may remain the only solution. Beyond representing a complication of implant-associated infections, repeated surgical interventions are associated with increased perioperative risk and cumulative physiological burden and may negatively affect patient outcomes, including overall survival, particularly in elderly and multimorbid patient populations [13,50,51]. Additionally, inflammatory conditions within the body, associated with other independent diseases, increase the risk of infection in immunosuppressed groups, highlighting the need for individualized selection of implant biomaterials for each patient [15]. Implants placed in the body may initiate surgical site infections (SSIs), which represent some of the most common and most dangerous complications following surgical procedures, particularly those involving implantation. Such infections occur when bacteria colonize tissues at the site of the surgical incision, leading to inflammation, abscess formation, or the formation of a biofilm in the presence of a foreign body (e.g., an implant) [52]. The site of biofilm formation depends both on the type of implant and its location within the body, as well as on the species of microorganism initiating the process (Table 1). The bacterial species most commonly encountered in the human body include *Escherichia coli* and *Staphylococcus aureus*. *Escherichia coli* is a natural component of the intestinal microbiota; however, disruption of microbial balance may lead to infections in other organs. In contrast, *Staphylococcus aureus* primarily colonizes the skin and mucous membranes and, in cases of tissue damage or the presence of implants, frequently causes local and systemic infections. Both species are among the most common causative agents of infections in humans [53].

#### 4.4.1. Biofilm in the Stomatognathic System

**Figure 3:**
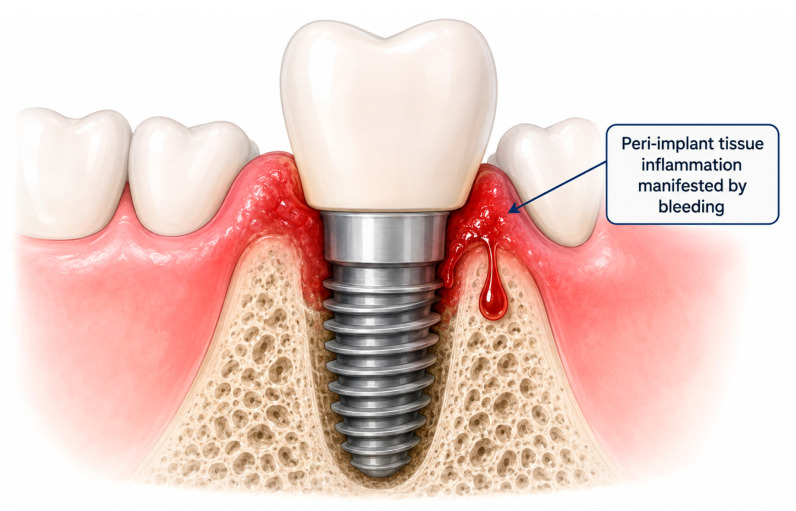
Peri-implant tissue inflammation manifested by bleeding.

A specific example of an implant-associated biofilm is peri-implantitis (from Greek peri, meaning “around,” and Latin implantitis, meaning inflammation of an implant), i.e., an inflammatory condition affecting both the gingiva and the bone surrounding a dental implant. This condition develops primarily as a result of biofilm colonization of dental implant surfaces. Untreated peri-implantitis may lead to bone loss and implant loosening, which may ultimately result in complete implant removal [50,55]. Oral biofilms have a composition similar to that of dental plaque and contain bacterial cells surrounded by a layer of exopolysaccharides, microorganisms, salivary components, and epithelial fragments. The presence of anaerobic pathogenic strains promotes the development of inflammation of the mucosa surrounding the implant, as well as other inflammatory conditions of the oral cavity associated with dental prostheses (Figure 3).

#### 4.4.2. Biofilm in the Skeletal System

**Figure 4:**
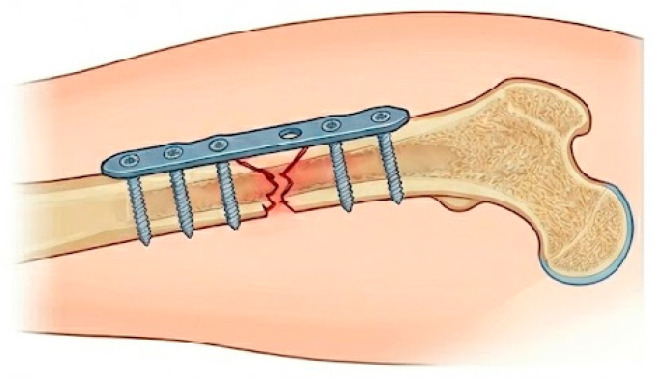
Infection occurring around a plate following fracture stabilization by internal fixation.

In the musculoskeletal system, biofilms accumulate on joint prostheses, particularly hip and knee ones (Figure 4). The formation of bacterial biofilms on the surface of plates used, for example, in the knee joint leads to intervention within the bone structure, which may result in osteomyelitis [56,57].

**Figure 5:**
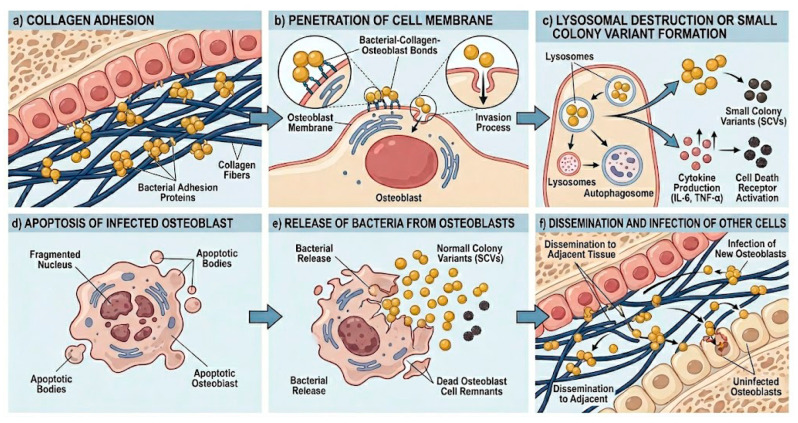
Process of osteomyelitis development induced by *Staphylococcus aureus*: (**a**) collagen adhesion; (**b**) penetration of the cell membrane through the formation of bacterial bonds with collagen molecules on osteoblasts; (**c**) possible destruction of bacteria present within osteoblasts by lysosomes isolated in vesicles, or the formation of small colony variants, followed by cytokine production and activation of cell death receptors; (**d**) apoptosis (cell death) of infected osteoblasts; (**e**) release of bacteria from osteoblasts; (**f**) dissemination of released bacteria and infection of other cells.

In both of the systems mentioned above, i.e., within the stomatognathic system as well as the skeletal system, the process of bacterial biofilm formation on the bone surface proceeds, in a similar manner, through bacterial aggregation, as illustrated in Figure 5. For example, colonization of an implant by *Staphylococcus aureus* may lead to the formation of a bacterial matrix, followed by the entry of pathogens into the bloodstream, resulting in the development of systemic infections such as osteomyelitis.

#### 4.4.3. Biofilm in the Urinary System

**Figure 6:**
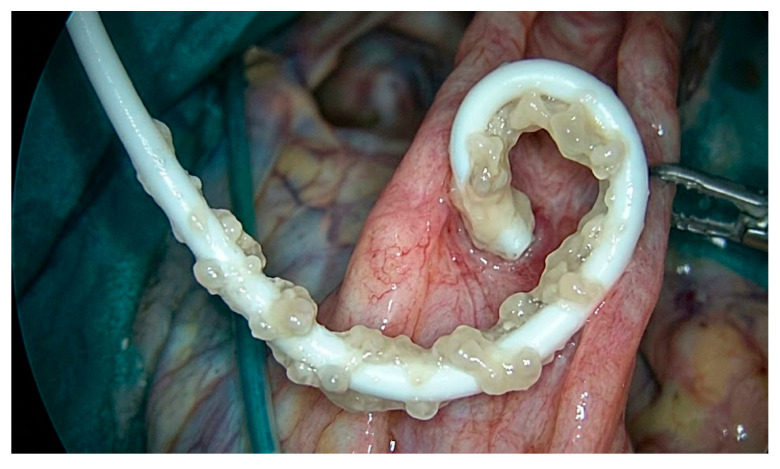
Heavily encrusted J-pigtail ureteral stent.

In the urinary tract, biofilms may colonize, among others, urological stents (Figure 6). In this case, contamination of the device most commonly occurs during its insertion, resulting in a urinary tract infection. The initial formation of biofilm on a catheter is associated with encrustation, which results from the crystallization of ionic components of urine as well as from biofilm formation, including the bacterial biofilm layer. The biofilm then gradually matures, forming a multilayered EPS structure that protects bacteria from the effects of antibiotics and disinfectants. In this way, hard deposits form on the surface of the stent, impairing urine flow and promoting chronic urinary tract infections [58,59].

**Figure 7:**
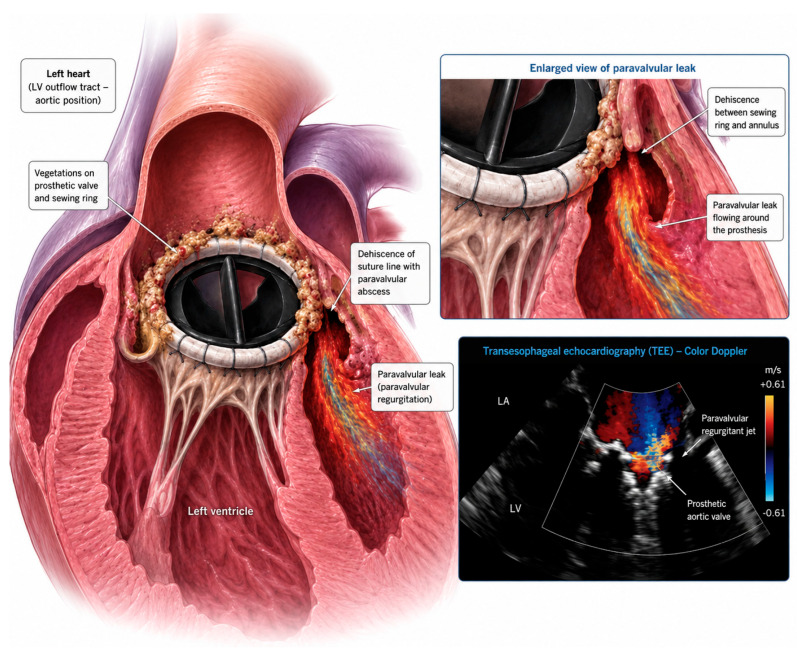
Endocarditis related to a prosthetic heart valve with a paravalvular leak.

In the cardiovascular system, biofilm formation is observed, among others, at sites of implantation of artificial heart valves and stents (Figure 7). Due to continuous blood flow, the presence of platelets and fibrin may induce aggregation and tissue damage, ultimately leading to the formation of fibrin clots. These areas are more susceptible to bacterial colonization, which may lead to the formation of bacterial aggregates and peri-implant inflammatory conditions, such as endocarditis or valve inflammation [17].

Each biofilm location is associated with a distinct set of microorganisms and specific microenvironmental conditions, influencing the dynamics of biofilm formation and resistance to treatment. These differences determine not only the microbiological composition of the biofilm but also its structure, growth rate, and susceptibility to external factors. For this reason, the effectiveness of methods aimed at inhibiting biofilm formation or eradicating biofilms depends on the site at which biofilms occur [60]. The following section discusses the most important strategies for combating bacterial biofilms. Biofilm formation is not limited to orthopedic and dental applications but is also highly relevant in cardiovascular implants (e.g., vascular grafts and stents), neurosurgical shunts, and cranial implants, where it contributes to device-related infections and long-term complications [5,6,9,51]. Across these applications, similar biofilm control strategies are employed, including antimicrobial surface modifications, release-based coatings, and anti-adhesive surface engineering approaches [17,18,61].

The colonization of implant surfaces by bacteria is a complex process determined by both the physicochemical properties of biomaterials and biological and environmental factors. From the perspective of microorganisms, this process offers significant adaptive opportunities, enabling them to achieve durable adhesion to the surface and form stable biofilm structures. Properties such as increased roughness, hydrophobicity, and high surface energy promote protein adsorption and the initiation of bacterial adhesion, leading to effective colonization and further biofilm development. At the same time, the presence of biofilms on implants is associated with numerous disadvantages in a clinical context. This process leads to the development of implant-related infections, including surgical site infections, peri-implantitis, and infections of the musculoskeletal and cardiovascular systems. These infections are often chronic in nature, potentially leading to tissue degradation, loss of implant function, and the need for its removal. Additionally, biofilms can serve as a source of systemic infections, increasing the risk of serious complications.

Diagnostic and therapeutic limitations also pose a significant problem. The structure of the biofilm, particularly the presence of an EPS matrix, significantly impedes drug penetration and limits the effectiveness of standard antibiotic therapy. Treatment efficacy depends on numerous factors, such as the type of biomaterial, the implant’s location, the microbiological composition of the biofilm, and the patient’s overall health. Additionally, factors such as compromised immunity, smoking, or perioperative conditions can promote the development of infection and limit the effectiveness of therapy. Despite these challenges, understanding the mechanisms of bacterial adhesion and biofilm formation has significant clinical applications. This knowledge is applied in the design of biomaterials with modified surface properties, the development of strategies to prevent bacterial colonization, and the optimization of treatment for implant-related infections. Of particular importance is the concept of the “race for surface,” which highlights the need for rapid integration of the implant with host tissues as a factor limiting biofilm development. In summary, interactions between implant surfaces and microorganisms play a key role in the pathogenesis of infections associated with biomaterials and represent a significant clinical challenge, requiring further research and the development of effective preventive and therapeutic strategies.

### 4.5. Clinical Impact of Biofilm-Associated Infections in Healthcare Settings

#### 4.5.1. Mechanisms of Biofilm Persistence and Antibiotic Tolerance

Bacterial cells forming the biofilm structure are isolated from the external environment and therefore acquire resistance to disinfectants and antibiotics. However, viable cells within the biofilm structure that remain metabolically inactive for a certain period after exposure to antibiotics retain the ability to become active again, allowing the biofilm structure to be re-established. The ability of biofilms to survive harsh external environmental conditions makes eradication, i.e., the process of biofilm removal, a significant challenge, thereby prompting the search for new solutions [12]. Based on their mode of action, biofilm combat methods can be classified into three categories: physical, chemical, and biological.

#### 4.5.2. Physical Strategies for Biofilm Removal

Physical methods include, among others, the use of extreme temperatures (both high and low), alternating magnetic fields (AMFs), and ultrasound [38]. The primary physical method that effectively leads to biofilm destruction is mechanical removal of the structure through surface cleaning, such as brushing or scraping. However, Daniluk et al. [62] demonstrated that a limitation of this method is its low versatility due to the diversity of substrate types. Another approach involves the application of high temperatures. Studies by O’Toole et al. [63] confirmed that biofilms exposed to temperatures in the range of 60–80 °C undergo complete destruction. Similarly, Ivaške et al. [64] applied low temperatures, using repeated freeze–thaw cycles at T = −45 °C, which resulted in effective biofilm eradication. The use of alternating magnetic fields for biofilm destruction involves the application of high-frequency fields (>100 kHz). Krishnamurthi et al. [65] confirmed that the eddy currents generated during field application enable the generation of heat, which can damage bacterial cell membranes and remove biofilm cells from the substrate surface. Bacterial membrane intensity increased following the exposure of bacteria to an electric current in the microampere range. Another approach involves the use of ultrasound to kill adherent bacteria before biofilm formation, as well as to combat already established bacterial structures. When ultrasound is applied, a high-intensity ultrasonic field forms and interacts with the implant surface, causing wave reflection and local mechanical effects. Transient cavitation, i.e., a cleaning procedure using ultrasound, is considered the most effective method for removing bacterial biofilms. When high intensities and low frequencies are applied, the opposite effect, namely, cell proliferation, does not occur. The result is the destruction of the EPS matrix and of the outer layer of the biofilm structure [61]. However, the effectiveness of the method has been demonstrated primarily in reducing the number of bacterial cells prior to adhesion, as demonstrated by Axelsson et al. [66].

#### 4.5.3. Chemical Approaches to Biofilm Control

Among the strategies employed, chemical methods include the use of synthetic poly(ethylene glycol) (PEG), chitosan (CS), and combinations of various therapeutic agents. These methods may affect the bacterial adhesion process as well as cell growth and division. Synthetic poly(ethylene glycol) (PEG) is the most commonly used agent and, as demonstrated in studies, reduces water activity in the cellular environment, inhibits the function of bacterial proteins, and limits further bacterial adhesion, which otherwise leads to biofilm formation. Ambrose et al. [67] confirmed that PEG, in the form of thin coatings or hydrogels, reduces the adhesion of bacteria such as *E. coli* and *S. aureus*. The application of thick, soft PEG hydrogels significantly reduced bacterial adhesion, an effect resulting from both decreased protein interactions and the physical properties of the surface. Effective biofilm eradication was achieved only through the combination of several agents to which microorganisms do not exhibit resistance. Jacqueline et al. [13] applied combination therapy involving moxifloxacin and daptomycin, which, when combined with clarithromycin, effectively eliminated *S. aureus* bacterial strains. The adhesive capacity of bacteria, which represents a key stage in biofilm formation, is also assessed using the Biofilm Ring Test. This method, based on the immobilization of magnetic beads within bacterial aggregates after biofilm formation in microplates, enables the measurement of cellular adhesion capacity. Chemical methods also include the use of chitosan, a linear polysaccharide that inhibits bacterial growth through positively charged amino groups interacting with the negatively charged bacterial outer cell membrane. The attachment of chitosan-containing hydrogels to surfaces aims at providing antibacterial activity and preventing further biofilm formation [64]. He et al. [68] developed a bilayer hydrogel coating capable of switching between a mode promoting cell adhesion and an antibacterial mode. In addition, the morphology of cells proliferating for 7 days on substrates coated with polymer brushes based on silica hydrogel, as well as on silica hydrogel substrates, was examined using SEM (scanning electron microscopy) and fluorescence microscopy. On the bare silicon (Si) plate, only individual cells with a rounded shape were observed, indicating that the surface did not promote cell spreading or growth. In contrast, on substrates coated with a bilayer coating, the cells formed an almost continuous layer with a characteristic spindle-like shape, spreading across the entire surface. It was also observed that cell density increased with increasing density of polymer brushes. Biological methods for combating biofilms most commonly include phage therapy, the use of ammonium salts, enzymes, and inhibition of quorum sensing. Among these approaches, phage therapy, involving the use of viruses attacking bacteria, appears to be the most effective. Phage therapy is particularly effective in eliminating multidrug-resistant bacteria. Międzybrodzki et al. [69] demonstrated phage activity against multidrug-resistant *S. aureus* strains. A promising approach also involves chemical compounds, such as quaternary ammonium salts (QAS), combined with maleic acid, used for the surface modification of cotton textile wound dressings. Li et al. [70] presented a modification of cotton fabric using maleic acid–based compounds derived from rosin acid. The resulting material exhibited strong antibacterial activity against both Gram-negative bacteria (*Escherichia coli*, *Pseudomonas aeruginosa*) and Gram-positive bacteria (*Staphylococcus aureus*). Importantly, the modified fabric significantly inhibited biofilm formation even after repeated contact with phosphate-buffered saline (PBS) solution and storage for 6 days, while maintaining good biocompatibility. *Staphylococcus aureus* and *Escherichia coli* cells were observed after contact with uncontaminated antibacterial cotton textiles (CT). In contrast, after exposure to fabric modified with quaternary ammonium cations of maleopimaric acid (CT-g-MPAN+), a significant reduction in the number of green and red fluorescent points was observed, indicating bacterial cell disruption or lysis without preservation of their integral morphology.

#### 4.5.4. Biological Strategies to Biofilm Control

Another method involves the use of enzymes degrading the EPS matrix, leading to weakened intercellular connections and reduced adhesion to the substrate surface. As a result, the biofilm loses its tolerance to environmental factors, and the cells become more sensitive to antibiotic treatment. Studies by Khan et al. [71] demonstrated that the application of glycosidase, protease, and DNase I significantly degraded the EPS matrix, compromising the mechanical stability of the biofilm and enabling more effective penetration of antibiotics into bacterial cells. Enzymes also play an important role in a method known as quorum quenching, which involves disruption of the standard quorum-sensing (QS) system, i.e., a mechanism that enables intercellular communication and is responsible for regulating gene expression. Blocking this process leads to degradation of the bacterial cell communication pathway and inhibition of further biofilm growth. Another approach involves the use of acidic electrolyzed water (AEW), which destabilizes the EPS matrix through cleavage of carbohydrate bonds, resulting in biofilm degradation. Okanda et al. [72] confirmed that AEW with a pH of approximately 5.5 effectively degrades the polysaccharides of the EPS matrix in *Pseudomonas aeruginosa* strains, thereby facilitating their removal. Similar effects are exhibited by phytochemicals, that is, natural plant-derived compounds, which can act as quorum-sensing inhibitors, limiting communication between bacterial cells. 

Natural compounds, such as berberine, also inhibit biofilm formation, as demonstrated by Zhou et al. [73,74]. The above-presented methods for combating biofilms include various approaches focused both on eliminating existing biofilm structures and on preventing their formation. However, their effectiveness may be limited by the specific implant environmental conditions as well as by the properties of the materials used in their fabrication. Therefore, a promising strategy for the prevention and treatment of biofilm-associated infections is the surface modification of implant materials, aimed at hindering bacterial adhesion or providing the surface with antimicrobial properties. This issue is discussed in detail in the subsequent chapters.

### 4.6. Clinical Consequences of Biofilm-Associated Infections

Biofilm-associated infections exhibit increased tolerance to antimicrobial therapy, which contributes to treatment failure, infection persistence, and a higher likelihood of surgical intervention [6,8,12]. The ability of bacterial biofilms to survive under adverse environmental conditions stems from their complex structure and the presence of cells with reduced metabolic activity, which allows them to adapt from the microorganisms’ perspective. These cells can survive exposure to antibiotics and subsequently become reactivated, leading to the restoration of the biofilm structure. Additionally, the extracellular matrix (EPS) provides an effective protective barrier, increasing the biofilm’s resistance to physical, chemical, and biological factors. At the same time, these properties translate into significant drawbacks in a clinical context, as biofilms exhibit high tolerance to standard treatment methods, making their complete eradication difficult. This results in the persistence of chronic infections and an increased risk of infection recurrence, even after antibiotic therapy. The limitations of available methods for combating biofilms are also a significant problem. Physical methods, such as mechanical removal, heat, ultrasound, or alternating magnetic fields, can be effective; however, their application is often limited by surface properties and the location of the biofilm. Chemical methods, including the use of compounds such as PEG or chitosan and combination therapies, exhibit antibacterial activity; however, their effectiveness depends on the appropriate selection of substances and the ability to penetrate the biofilm structure. Biological methods, on the other hand, including phage therapy, the use of enzymes that degrade the EPS matrix, or the inhibition of quorum sensing, represent a promising approach, but their effectiveness may be limited by the specific nature of the environment and the variability of microorganisms. Despite these challenges, the strategies being developed to combat biofilms have significant clinical applications, particularly in the prevention and treatment of infections associated with implants and chronic wounds. The use of a combination of physical, chemical, and biological methods, as well as the development of new materials with antibiofilm properties, enables an increase in the effectiveness of therapy. A particularly promising direction is the modification of biomaterial surfaces to reduce bacterial adhesion and enhance the activity of antimicrobial agents. In summary, effective biofilm control requires a multifaceted approach that accounts for both its complex structure and environmental variability, which represents one of the key challenges of modern medicine.

## 5. Issues Related to the Use of Titanium Alloy Implants

Due to the difficulties associated with combating biofilms, the selection and properties of materials used in implantology play an increasingly important role. Among the available biomaterials, titanium alloys are of particular importance. Due to their favorable mechanical properties, high corrosion resistance, and good biocompatibility, these materials are widely used in medicine. However, despite numerous advantages, bacterial adhesion and biofilm formation may also occur on their surfaces, posing a serious threat to the success of the implantation process. Currently, the medical industry requires the use of biomaterials that simultaneously exhibit appropriate mechanical as well as physical and chemical properties [23]. In addition, such materials should be characterized by low density, high static and fatigue strength, resistance to fracture, and high corrosion resistance. It is estimated that approximately 70–80% of implants are made from metallic biomaterials, of which titanium (Ti) and its alloys account for about 40% of the biomaterials used for implants. They have found widespread application in orthopedics and prosthetics due to their favorable mechanical properties, corrosion resistance (higher than that of 316 L stainless steel or Co–Cr–Mo alloys), high fatigue strength, and an elastic modulus close to that of human bone (approximately E = 79 GPa). Another advantage is the retention of functionality even in the presence of bodily fluids [24,25]. The most commonly used biomaterial in this group is the Ti–6Al–4V alloy, used for the fabrication of prosthetic components and implants. Despite its widespread use, it has certain disadvantages, including low biological activity (which limits fibrous tissue growth and promotes prosthesis loosening), the risk of metallosis, and associated inflammatory conditions or other allergic reactions. Additionally, adverse effects of vanadium, such as cytotoxicity, may result in damage to the nervous system or lead to the development of Alzheimer’s disease or even cancer [43]. Similar concerns have been raised regarding the presence of aluminum (Al), prolonged exposure to which in tissues may also have toxic effects. These implant-related requirements have driven the development of titanium alloys, such as Ti–6Al–7Nb.

### New-Generation Titanium Alloys

New generations of vanadium-free titanium alloys were developed to eliminate the harmful effects of vanadium and aluminum. Initially, modifications involved replacing vanadium with niobium, as in the Ti–6Al–7Nb alloy; however, even in this case, the presence of aluminum was associated with a risk of adverse effects on the body. In subsequently developed titanium alloys, vanadium and aluminum were replaced with biologically inert elements such as zirconium (Zr), niobium (Nb), tantalum (Ta), and molybdenum (Mo). One of the most promising titanium alloys of this generation is Ti–13Nb–13Zr, which contains only vital elements that do not induce inflammatory responses or allergic reactions [75,76] (Table 2).

The vital elements present in the chemical composition of the Ti–13Nb–13Zr alloy, such as niobium (Nb) and zirconium (Zr), are non-toxic, improve biocompatibility, and do not exert negative effects on tissues or the human body, providing an advantage over the conventional Ti–6Al–4V alloy. The modification of the chemical composition also results in increased corrosion resistance, attributable to the high stability of passive layers and the low dissolution rate in physiological fluids [16,75]. It has been demonstrated that the Ti–13Nb–13Zr alloy maintains passivity under simulated physiological conditions, such as Ringer’s solution, artificial saliva, and phosphate-buffered saline (PBS), whereas its electrochemical activity is observed in hydrochloric acid solutions. Other studies indicate that alloys based on the Ti–Nb–Zr system are potentially more resistant to environmental factors in orthopedic applications than in dental applications. Interest in these alloys arises primarily from the needs of joint alloplasty, where osseointegration, defined as the direct interaction between a metallic implant and bone, plays a crucial role. The smooth passive layer of titanium dioxide (TiO_2_), which naturally forms on the surface of titanium, does not always provide sufficient bioactivity and may lead to the formation of fibrous tissue instead of a stable bond with bone, thereby increasing the risk of implant loosening and inflammation. Continuous research focused on improving the properties of implant surface layers and enhancing osseointegration enables the development of techniques and methods for modifying coatings and surface layers. The search for new solutions has led to the development of novel Ti–Nb–Zr and Ti–Nb–Sn systems, as well as the introduction of alloying elements such as tantalum (Ta) and molybdenum (Mo), which are particularly important in medical applications. These materials find potential applications, among others, in bone screws, plates, and intramedullary nails. Currently, research is conducted on the use of Nb- and Zr-containing alloys in the design of hip and knee joint endoprostheses as long-term implants. The Ti–13Nb–13Zr alloy is classified as a β-type alloy, although some studies indicate that it only approaches this region [44]. It is characterized by a lower β-transus temperature during heating, equal to T = 735 °C, compared with the commonly used Ti–6Al–4V and Ti–6Al–7Nb alloys. In this case, the addition of Nb, acting as a β-phase stabilizer, lowers the martensitic transformation start temperature, as a result of which the Ti–13Nb–13Zr alloy is characterized by a Young’s modulus close to that of human bone [80]. In addition, it exhibits superior mechanical properties compared with conventional titanium alloys, making it one of the most promising alternatives in implantology (Table 3).

Zirconium (Zr), as a neutral element, does not significantly affect phase transformation temperatures but provides solution strengthening of both the α and β phases. Common alloys containing additions of niobium or vanadium exhibit Young’s modulus values in the range of approximately E = 100–110 GPa [81,82]. Numerous studies conducted on the Ti–13Nb–13Zr alloy, focusing on its microstructure, hardness, tribological behavior, and corrosion resistance, highlight the growing interest in this biomaterial. Gołasz et al. [83] and Acimert et al. [84] have shown that optical profilometry and atomic force microscopy (AFM) are the most commonly used methods to determine surface roughness parameters and to assess surface wettability. Evaluation of uncoated and unmodified samples indicates that unmodified Ti–13Nb–13Zr exhibits a range of hydrophilicity from very high (angle ~26°) to moderate (~48–83°), depending on the measurement scale and surface preparation (differences in polishing/grinding procedures and instrumentation). Surface roughness ranges from tens of nanometers (AFM) to approximately 0.6–1.9 µm (profilometry); higher roughness promotes increased wettability (lower angles), which is beneficial for cell adhesion. However, there are still no comprehensive analyses addressing its antibacterial properties, potential ion release from the alloy surface, and the influence of an environment simulating physiological conditions on surface stability. Both the research group of Łukaszyk et al. [85] and many other authors [82,83] focused primarily on the physicochemical characteristics of the alloy, without extending their studies toward antibacterial activity. The evaluation of the Ti–13Nb–13Zr alloy using methods such as XRD (X-ray diffraction), SEM (scanning electron microscopy), and XPS (X-ray photoelectron spectroscopy), as well as corrosion resistance, cytotoxicity, and mechanical property tests, provides a solid basis for comparison with conventional titanium materials used in implantology. However, comprehensive studies addressing bacterial adhesion and ion release are still lacking [75]. To date, analyses addressing biofilm formation and cytotoxicity have been conducted primarily for the Ti–6Al–4V alloy, whereas analogous studies for Ti–13Nb–13Zr remain limited [86]. Available results nevertheless indicate that the surface of the Ti–13Nb–13Zr alloy exhibits the strongest biofilm-inhibiting properties in relation to *Enterococcus faecalis*, *Pseudomonas aeruginosa*, and *Staphylococcus aureus*, outperforming the Ti–6Al–4V alloy in this respect. Moreover, despite the high biocompatibility of Ti–6Al–4V, the Ti–13Nb–13Zr alloy promotes osteoblast differentiation to a greater extent, which is critical for applications in bone surgery [16]. Studies of *S. aureus* culture on the surfaces of both Ti–6Al–4V and Ti–13Nb–13Zr samples, conducted for 24 h, showed significantly lower expression of genes associated with biofilm formation on the Ti–13Nb–13Zr alloy. In addition, images obtained using scanning electron microscopy confirmed reduced bacterial colonization on the surface of the Ti–13Nb–13Zr alloy after 24 h of incubation. These results indicate improved resistance in the context of implant-related applications and a potential reduction in the risk of infection [87].

Studies have shown that the permeability and release of ions from the Ti–13Nb–13Zr alloy are significantly lower than those from pure titanium (cp-Ti) or the Ti–6Al–4V alloy, which is directly related to the β-type structure. This indicates that β-type alloys (Ti–Nb–Zr) exhibit a lower tendency toward ionization and the release of biologically unfavorable ions compared with traditional alloys containing vanadium and aluminum [88]. At the same time, the reduced permeability and release of ions minimize the risk of cytotoxicity or inflammation in the surrounding tissue. Analyses conducted for pure titanium and the Ti–13Nb–13Zr alloy subjected to high-pressure torsion revealed differences in ion release rates compared with the unmodified material. However, there are still no comprehensive studies addressing both the long-term kinetics of ion release and the effects of released ions on the body in relation to the physicochemical properties of this new-generation alloy [89]. This highlights the need for further analyses of the Ti–13Nb–13Zr alloy, particularly with respect to its antibacterial properties and long-term biocompatibility. Although the Ti–13Nb–13Zr alloy exhibits superior antibacterial properties and higher biocompatibility than previously used titanium alloys containing vanadium or niobium, its intrinsic antibacterial activity remains insufficient. For this reason, increasing attention is being directed toward advanced surface modification methods that can significantly reduce the risk of bacterial colonization. One of the most promising approaches involves the use of bactericidal and bacteriostatic coatings forming an active antimicrobial barrier. The following section of this thesis discusses available surface modification methods and the characteristics of coatings used in the prevention of biofilm formation on implants. The search for optimal combinations of material properties is associated not only with the development of new alloys but, above all, with the improvement of surface layers [25].

Methods for combating biofilms include physical, chemical, and biological strategies, which differ in their mechanisms of action, efficacy, and scope of application. Their main advantages include a broad spectrum of action, ranging from the direct destruction of the biofilm structure (e.g., through heat, ultrasound, or electromagnetic fields) to inhibiting bacterial adhesion and growth (e.g., using PEG or chitosan) to more targeted biological methods such as phage therapy, the use of enzymes, or quorum-sensing inhibition. The use of combination therapies is particularly important, as they enhance the effectiveness of biofilm eradication and limit the development of microbial resistance. Additionally, some approaches, especially enzymatic ones, enable the degradation of the EPS matrix, which improves antibiotic penetration and increases their efficacy. Despite these advantages, the methods discussed also have significant limitations. Many of them are not sufficiently effective as standalone solutions and require a combination with other therapeutic strategies. Physical methods may lack selectivity and lead to tissue damage or damage to implant surfaces, while chemical methods may affect the properties of biomedical materials and have a limited duration of action. In the case of biological methods, challenges remain regarding their stability, control of action, and often high specificity toward specific bacterial strains. An additional problem is the high resistance of biofilms resulting from the presence of cells in a state of metabolic dormancy and the protective role of the extracellular matrix, which significantly hinders their complete elimination. Furthermore, the effectiveness of individual methods is strongly dependent on environmental conditions, such as the type of surface, the presence of body fluids, or the degree of biofilm maturity. The lack of a universal strategy effective against all types of biofilms makes a multidirectional approach necessary. In clinical practice, the described methods are primarily used in the prevention and treatment of infections associated with medical implants. They are employed both for surface disinfection and for modifying implant materials, with the aim of reducing bacterial adhesion and preventing biofilm formation. Combined strategies and advanced surface modification technologies are gaining increasing importance, as they enable the incorporation of antimicrobial properties into implants as early as the design stage.

## 6. Characteristics of Bactericidal and Bacteriostatic Coatings

The development of functional coatings on titanium implant surfaces not only aims at improving osseointegration but also at significantly reducing the risk of infections associated with bacterial biofilm formation. A response to the growing challenges posed by bacterial resistance to antibiotics involves the development of coatings that act directly at the implant–tissue interface, exhibiting bactericidal or bacteriostatic properties. Such coatings may contain active components, such as metal ions (e.g., silver (Ag), copper (Cu), and zinc (Zn)), organic compounds such as antimicrobial peptides, or hybrid biomaterials that simultaneously support integration with bone tissue. Antimicrobial coatings are deposited onto surfaces in order to inhibit bacterial growth and proliferation through the release of active components. Silver (Ag) remains one of the most commonly used elements due to its strong bactericidal activity, primarily attributed to the release of Ag^+^ ions. The binding of silver ions to bacterial proteins and nucleic acids leads to destabilization of the cell membrane, damage to enzymatic proteins, and disruption of DNA replication. In addition, silver nanoparticles enhance the generation of reactive oxygen species in the immediate vicinity of bacteria, resulting in reduced bacterial viability at relatively low doses [90]. The most commonly used approaches include coatings incorporating Ag^+^ ions and coatings containing antibiotics. Ag^+^ ions interact with proteins and DNA, destabilizing the cell membrane and intracellular structures, ultimately leading to the death of bacteria. Ag^+^-releasing coatings effectively inhibit bacterial growth. An example is TiO_2_ nanotubular layers with Ag nanoparticles, exhibiting bactericidal activity against *Staphylococcus aureus* while maintaining high biocompatibility [91]. Similar effects have been achieved with gelatin-releasing coatings in composite systems supporting bone tissue regeneration [61]. Another commonly used bactericidal element is copper (Cu). It acts through direct damage to the cell membrane, protein destabilization, and the generation of reactive oxygen species, as well as through the catalysis of reactions. Copper ions exhibit rapid bactericidal activity against typical pathogens associated with peri-implant infections, while at appropriate concentrations they may simultaneously support the proliferation and differentiation of bone cells. For this reason, both Cu-ion–releasing coatings (e.g., through doping of TiO_2_ or Ti–Cu alloys) and contact coatings (e.g., Ti–Cu alloys) have been developed, providing long-term antibacterial effects while maintaining high biocompatibility [92]. Moreover, zinc (Zn) and its oxides combine antibacterial activity with beneficial effects on osteoblasts and stem cells, improving osseointegration. Zinc ions also inhibit certain bacterial enzymes, which affects membrane growth and may interact with the generation of reactive oxygen species, while simultaneously contributing to enhanced osteoblast proliferation and differentiation. However, due to the cytotoxicity of zinc at high concentrations, the search for more biocompatible elements is ongoing [93]. Other approaches include hydroxyapatite (HA) and titanium oxide coatings applied by plasma spraying [94], composite layers made from polycaprolactone (PCL) and bioactive glass with the addition of silver and graphene [18] or Cu, Zn, and Mg ion implantation [19]. Hydroxyapatite, a mineral component similar to bone tissue, improves implant integration. Moreover, it has been demonstrated that Cu exhibits bactericidal activity against *Staphylococcus aureus* with an effectiveness exceeding 99%, while Zn and Mg additionally support osteoblast proliferation and cell differentiation. Superhydrophobic coatings obtained by anodization on titanium surfaces (wetting angle > 170°), developed by Rao et al. [95], reduced bacterial adhesion by approximately 98%. Surface modification was performed using ultrasonic treatment with 1H,1H,2H,2H-perfluorodecyltriethoxysilane (FAS). Recent years have seen an increasing interest in tin and its oxides as alternative coating materials. Tin exhibits antibacterial activity but is also characterized by relatively low cytotoxicity compared with silver or copper. Consequently, tin is a promising material for the design of modern implants. Tin oxide (SnO_2_) is a semiconducting metal oxide characterized by photocatalytic properties, high chemical stability, and relatively low toxicity. Tin(IV) oxide, used in the form of nanofibers, thin-film coatings, or composites with other metal oxides (e.g., TiO_2_ or RuO_2_), reduces bacterial adhesion, disrupts biofilms, and may simultaneously stimulate stem cell differentiation, supporting osseointegration. Appropriately designed SnO_2_ structures may exhibit passive effects resulting from their surface properties or active behavior by responding to stimuli such as light or electrical impulses. Zhou et al. [96] described a multilayer TiO_2_–SnO_2_–RuO_2_ coating responsive to electrical stimuli, eliminating bacteria and simultaneously increasing the expression of osteogenic genes. The electrically charged surface effectively eliminated bacteria colonizing the implant and simultaneously supported integration with bone tissue, which translates into increased implant durability. Other studies have confirmed the high biocompatibility of SnO_2_ coatings (>90% cell viability), their activity against six bacterial strains, and strong antibacterial properties resulting from the photocatalytic activity of SnO_2_ [97]. Sn nanoparticles reduce the adhesion and proliferation of *S. aureus* without adversely affecting biocompatibility. Such modifications make it possible to increase implant durability and reduce the risk of complications and the need for reoperation [20]. In summary, modifications of implant surfaces using antibacterial elements represent an important direction in the development of biomaterials. They combine biological functionality with antibacterial protection, thereby enhancing the effectiveness of implant-related treatments. However, a key aspect remains the selection of an appropriate coating deposition method, as it determines both the durability and biological activity of the coatings.

The development of functional coatings on the surface of titanium implants is currently focused not only on improving osseointegration but also on effectively reducing the risk of infections associated with bacterial biofilm formation. The response to the growing problem of bacterial resistance to antibiotics is the design of coatings that act directly at the implant–tissue interface, exhibiting bactericidal or bacteriostatic properties. Their main advantages include the ability to locally release active components, such as metal ions (e.g., Ag, Cu, Zn), antimicrobial peptides, or hybrid materials that simultaneously support bone tissue regeneration. Coatings containing silver are particularly widely used; their mechanism of action is based on the release of Ag^+^ ions that destabilize bacterial cell membranes, damage enzymatic proteins, and disrupt DNA replication. Additionally, silver nanoparticles enhance the generation of reactive oxygen species, which increases the effectiveness of the antimicrobial action even at low concentrations. Coatings containing copper exhibit similar properties; copper acts by damaging cellular structures and inducing oxidative stress, and at appropriate concentrations, it can simultaneously support the proliferation and differentiation of bone cells. Zinc, on the other hand, combines antibacterial activity with a positive effect on osteoblasts and stem cells, which promotes osseointegration. A key advantage of these solutions is the ability to achieve both antibacterial and bioactive effects simultaneously, making them particularly attractive for clinical applications. The use of materials such as hydroxyapatite, bioactive glasses, or polymer composites further enhances the integration of the implant with bone tissue. Modern strategies also include modifications to surface topography, such as superhydrophobic coatings that limit bacterial adhesion, or the use of semiconductor materials (e.g., SnO_2_), which, thanks to their photocatalytic properties, can actively reduce the presence of microorganisms. Particularly promising are multilayer systems and coatings that respond to external stimuli, such as light or electrical impulses, enabling controlled antibacterial action while simultaneously supporting osteogenesis processes. Despite numerous advantages, these solutions are not without drawbacks. A key concern remains the potential cytotoxicity of metal ions at excessively high concentrations, particularly in the case of zinc or silver. Furthermore, the effectiveness of the coatings depends on the kinetics of active substance release, which in practice can be difficult to precisely control. For some systems, there is also a risk of limited coating durability or loss of their properties during implant use. An additional challenge is ensuring a balance between antimicrobial activity and biocompatibility so as not to disrupt tissue healing and regeneration processes. Another limitation is the lack of universal solutions that would be effective against a broad spectrum of pathogens while remaining completely safe for host cells. The long-term effects of using coatings, including their stability, impact on the biological environment, and potential immune reactions, still require further research. Another important aspect is the selection of an appropriate coating application method, which determines their physicochemical properties, durability, and biological activity. In the context of clinical applications, antimicrobial coatings are widely used in dental and orthopedic implantology, where preventing peri-implant infections and ensuring durable osseointegration are of critical importance. Modern approaches, combining bioactive and antibacterial properties, contribute to increased treatment efficacy, reduced risk of complications, and a lower need for reoperation. The development of smart coatings that respond to environmental stimuli represents one of the most promising directions in the design of next-generation implants.

### 6.1. Methods for Surface Modification of Titanium Biomaterials

**Figure 8:**
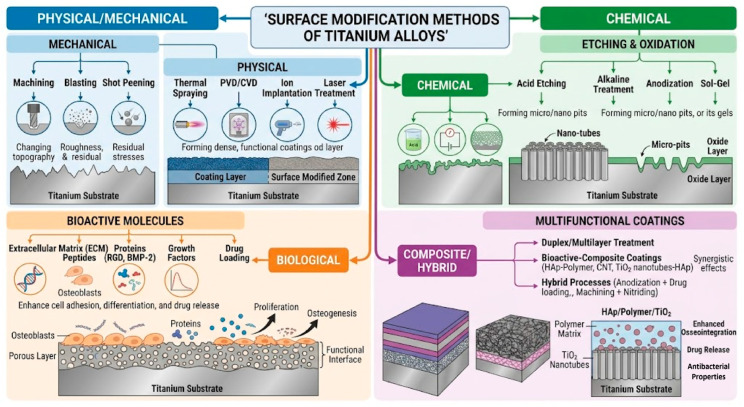
Surface modification methods.

The continuous development of biomaterials involves not only the design and surface processing of materials most commonly used for implant fabrication but also their further improvement through various modification strategies. These modifications play a crucial role in enhancing physicochemical properties, promoting osseointegration, and reducing the risk of peri-implant infections associated with both bacterial accumulation and the migration of ions from metals. As a result, modifications reduce the likelihood of bacterial biofilm formation [11,98]. Improving the surface of a biomaterial with respect to all these properties enables enhanced biocompatibility, which is of fundamental importance for the success of implant-based treatment. It is precisely the implant surface, remaining in direct contact with tissue, that determines the effectiveness of osseointegration and durability. The effectiveness of an implant, therefore, depends on its ability to support the process of bone integration as well as implant resistance to microbial colonization [21,22,99]. Despite the fact that titanium alloys are widely recognized as biomaterials with very good biocompatibility, their surfaces exhibit biological inertness, which makes them susceptible to bacterial infections. Moreover, these alloys may exhibit insufficient mechanical stability and poor initial stability at the time of implant placement in bone. Consequently, advanced surface modification methods are gaining increasing importance, as they pose a challenge to conventional implant processing techniques [100]. Currently applied methods for the surface modification of titanium implants are aimed at improving osseointegration and reducing the risk of infection. These methods are divided into four main groups (Figure 8):

- Biological;
- Physical and mechanical;
- Composite;
- Chemical.

The selection of an appropriate implant surface modification method depends on many factors, such as the site of implant application and the required physicochemical or biological properties. The integration of advanced surface modification techniques enables a significant improvement in the performance and durability of metallic implants [100,101].

#### 6.1.1. Biological Methods of Surface Modification

Biological methods include surface functionalization with biomolecules such as peptides, proteins, or growth factors, whose role is to stimulate cellular responses and support osseointegration. The use of biologically active coatings, such as hydroxyapatite (HA), bioglass, or antimicrobial substances, improves implant integration with bone, reduces the risk of infection, and enhances biocompatibility. A modern solution involves composite coatings with a gradient structure; they combine various functions, for example, antibacterial activity with the stimulation of bone tissue growth. Among the most commonly used are antibacterial, polydopamine-based, and bioactive organic coatings. They form tissue-like structures, support cell adhesion, inhibit bacterial proliferation, facilitate bone tissue growth and integration, and enhance the biocompatibility and bioactivity of the implant. Such modifications play a key role in initiating surface changes at the micro- and macro-scale, which facilitate the formation of a stable bond between the implant and the surrounding bone tissue [100,101]. Physical and mechanical methods of implant surface modification include sandblasting, shot peening, surface coating, and laser surface enhancement (LSE). The aim of these techniques is to improve biocompatibility, osteoconductivity, and resistance to wear and fatigue and ensure the long-term stability of implants. Chang et al. [102] demonstrated that the combination of sandblasting and acid etching increases the hydrophilicity and roughness of titanium, thereby improving its surface properties. The applied coatings, most often biologically active, promote the adhesion and growth of bone cells. Fouda et al. [103] have shown that coating titanium implants with hydroxyapatite (HA) accelerates the healing process and enhances bonding with tissue; however, various deposition techniques may affect the strength and stability of the coatings. In some cases, coatings are susceptible to delamination, which may trigger an inflammatory condition. Laser surface enhancement (LSE) involves the selective melting or modification of the surface structure using a laser, without compromising the properties of the substrate. Arthur et al. [104] have demonstrated that, in the case of 3D-printed titanium alloy implants, this method improves corrosion resistance and mechanical properties. Compared with chemical and biological methods, physical and mechanical techniques may, however, lead to excessive surface roughness, thereby weakening the strength of interphase bonds. Their impact on biological activity is largely limited to improvements in mechanical properties achieved through modifications of surface topography.

#### 6.1.2. Multifunctional Coating of Surface Modification

**Figure 9:**
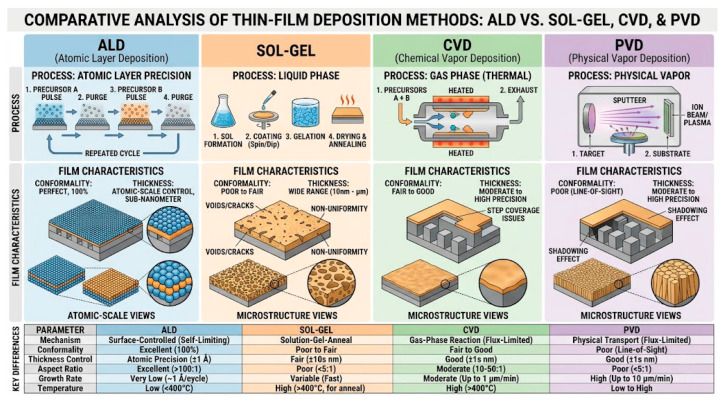
ALD method-based layer deposition compared with the sol–gel, CVD, and PVD methods.

The composite coating approach involves combining two or more methods in order to integrate their advantages and compensate for the limitations of individual techniques. Typical examples include composite coatings based on titanium oxide, hydroxyapatite (HA), and other bioactive materials. Multilayer structures enable the combination of functions provided by individual layers, enhancing mechanical properties, corrosion resistance, and antibacterial activity. Pretreatment processes, such as alkaline treatment, improve coating adhesion, while heat treatment reduces interlayer stresses, thereby stabilizing the structure. However, excessively thick coatings may be prone to delamination. Song et al. [105] combined alkaline treatment, heat treatment, and electrochemical HA deposition, resulting in the obtainment of implants with improved stability, bioactivity, and enhanced osseointegration. Zhang et al. [106] used selective laser melting (SLM) technology to fabricate porous scaffolds from a titanium alloy. The SLM process was followed by sandblasting, acid etching, and atomic layer deposition (ALD) of tantalum oxide, leading to the obtainment of uniform coatings improving cell adhesion, proliferation and differentiation of stem cells, as well as cytocompatibility and osseointegration. Chemical methods involve treating implant surfaces with chemical compounds in order to induce reactions and modify their properties. The most commonly used techniques include acid etching, anodic oxidation, micro-arc oxidation (MAO), electrophoretic deposition (EPD), physical vapor deposition (PVD), chemical vapor deposition (CVD), and its variant, i.e., atomic layer deposition (ALD). These methods improve biocompatibility and promote osseointegration. Acid etching (using HF, HNO_3_, H_2_SO_4_, or their mixtures) leads to the formation of microcavities, increasing surface roughness, thereby facilitating the adhesion and growth of bone cells. Attar et al. [107] demonstrated that this technique promotes macrophage adhesion and reduces levels of reactive oxygen species (ROS). A reduction in ROS levels translates into a lower risk of oxidative stress, which promotes favorable biological processes, such as improved implant healing and enhanced bone cell growth. Acid etching is often combined with anodization, which employs an electrochemical system and leads to changes in surface morphology as well as increased bioreactivity. Anodic oxidation is a technique that leads to the formation of a layer with controllable thickness, morphology, and crystalline structure, in which the implant serves as the anode in an electrolytic system. El-wassefy et al. [108] demonstrated that anodized surfaces exhibit increased bioactivity. Calcium phosphate layers produced using this method were characterized by improved mechanical properties of the implant–bone interface, enabling earlier implant loading. Micro-arc oxidation (MAO) is an electrochemical method that enables the formation of active oxide coatings, also on complex implants fabricated using 3D-printing technologies. Kozelskaya et al. [109] showed that this process enables controlling the thickness of porous coatings and enhances the biological activity of materials. Electrophoretic deposition (EPD) involves the deposition of charged particles in an electric field at low temperatures. This method enables the fabrication of uniform coatings with controlled thickness, most commonly composed of hydroxyapatite, graphene oxide (GO) and silver (Ag). Juliadmi et al. [110] applied EPD for coating of implants with hydroxyapatite, achieving improved surface properties. Chemical vapor deposition (CVD) is based on chemical reactions in the gas phase at high temperatures, enabling the precise fabrication of thin coatings, including HA coatings, supporting bone tissue growth. Rifai et al. [111] obtained diamond coatings using the CVD method. These coatings enhanced cell proliferation and inhibited bacterial growth. In recent years, atomic layer deposition (ALD) technology has gained particular importance. This method enables the deposition of ultrathin coatings with precisely controlled thickness, composition, and uniformity. Due to its ability to deposit uniform layers on surfaces with complex geometry and high porosity, this technique is widely used in the modification of titanium implants applied in orthopedics and stomatology. The ALD method enables the formation of nanoscale layers that uniformly coat biomaterials characterized by complex geometries. As a result, it is possible to precisely control coating growth mechanisms and coating continuity, which represents an advantage over other surface modification techniques (Figure 9) [112].

#### 6.1.3. Surface Modification by ALD Method

Compared with other coating deposition techniques, the ALD method is distinguished by the ability to precisely control coating thickness solely through the number of cycles performed, independent of precursor flux uniformity, which constitutes a limiting factor in methods such as CVD or PVD. An additional advantage is the ability to use highly reactive precursors, as the reactants come into contact only at the substrate surface, enabling immediate layer formation without the risk of uncontrolled growth. As a result, each cycle produces a strictly defined layer with nanometer-scale thickness. Compared with other thin-film deposition methods, ALD is distinguished by the precise control over the number of cycles. One cycle delivers a defined and repetitive amount of precursor, regardless of irregularities in the precursor flux or the geometry of the substrate. The ALD method is particularly valuable for the modification of biomaterials with porous and complex geometrical structures, as it enables uniform and precise deposition of thin layers even on hard-to-reach surface regions. This feature is particularly important and gives ALD an advantage over CVD and PVD in the case of porous and three-dimensional printed implants, where uniform coverage of hard-to-reach areas and internal channels is required [113]. This is also associated with the manner in which the coating is formed at the substrate–gas interface. The resulting coatings are bonded to the substrate via chemical bonds and, therefore, are characterized by high adhesion compared with coatings formed using other deposition methods. Moreover, the ALD method ensures controlled growth of the coating thickness by increasing the number of cycles during the process, thereby affecting the deposition of successive layers on the surface. The application of ALD enables not only improved biocompatibility but also provides surfaces with antibacterial properties, which is of key importance in the prevention of peri-implant infections. The technology makes it possible to deposit thin layers of metal oxides (e.g., TiO_2_ and ZrO_2_) as well as silver nanoparticles (Ag) without affecting the structure of the implant itself.

In this way, the ALD method enables the combination of several functional strategies [114]:

- Direct deposition of a thin layer with controlled thickness or amount of nanoparticles, enabling antibacterial activity;
- Deposition of active oxides with photocatalytic activity, which, under the action of selected factors (e.g., light, plasma, or electric current), generates reactive oxygen species destroying bacterial cells;
- Surface modification through changes in surface energy, surface wettability characteristics, or nanoscale topography, which, in the context of biofilm formation, reduces bacterial adhesion and initial growth.

Studies confirm the effectiveness of this method Devlin-Mullin et al. [114] deposited a silver layer on three-dimensional titanium implants, achieving a significant reduction in *Staphylococcus epidermidis* colonization while simultaneously supporting angiogenesis (the formation of new blood vessels—capillaries—from pre-existing vessels) and osseointegration in an in vivo model. Importantly, no signs of cytotoxicity were observed. Similarly, Nazarow et al. [115] obtained a silver nanoparticle-containing TiO_2_ coating characterized by a strong antimicrobial effect against *Staphylococcus aureus* strains and enhanced osteogenic activity as well as mesenchymal stem cell differentiation (MSC). The coating exhibited the ability to support osteoblast differentiation even in the absence of growth factors in the medium. Another approach involved the use of zirconium dioxide (ZrO_2_) deposited on the titanium surface using the ALD method. ZrO_2_ coatings, particularly in the tetragonal phase (i.e., within the temperature range of 1170–2370 °C), demonstrated the ability to reduce bacterial adhesion and improve osteoblast viability, thereby representing an alternative to silver-containing coatings. Zhao et al. [26] analyzed the effect of the number of ALD cycles for TiO_2_ coatings, showing that an increase in the number of cycles led to reduced surface wear and decreased emission of metal ions, which may limit inflammatory responses. In turn, Konopatsky et al. [27] applied ALD to modify the advanced titanium alloy Ti–18Zr–15Nb by depositing silver nanoparticles. The obtained surfaces were characterized by improved antibacterial properties while maintaining high biocompatibility, indicating the potential applicability of this type of surface modification also in implantology involving the use of next-generation biomaterials. In addition to surface modification techniques, physical metallurgy approaches have also been explored to enhance the antibacterial performance of titanium-based biomaterials. Alloying titanium with elements such as silver, copper, and zinc can introduce intrinsic antibacterial activity through controlled ion release and localized disruption of bacterial cell membranes, while simultaneously influencing corrosion resistance and mechanical properties [16,23,24,25,92,93]. Furthermore, the microstructural design of titanium alloys, particularly metastable β-type systems such as Ti–Nb–Zr-based alloys, plays a key role in tailoring both biological and functional properties of implants [75,76,81,82,116]. Such microstructural engineering may contribute to long-term performance through electrochemical interactions, including localized galvanic effects, which can further influence bacterial adhesion and biofilm development [16,44,80,83]. However, it is important to note that bulk modification strategies require careful balancing of antibacterial efficacy with mechanical integrity, corrosion resistance, and overall biocompatibility. Although atomic layer deposition (ALD) is widely recognized as a highly precise technique for fabricating uniform and conformal antibacterial coatings, including SnO_2_-based films, several inherent limitations should be explicitly considered. One of the main drawbacks is the intrinsically slow deposition rate associated with the self-limiting surface reactions, which restricts process scalability and industrial throughput. In addition, the functional performance of SnO_2_ coatings may be affected by energy-level alignment and band structure mismatches at the coating–substrate interface, which can influence charge transfer processes relevant to antibacterial mechanisms, particularly in catalytic or photoactive environments. Furthermore, depending on the precursor chemistry employed for SnO_2_ deposition, corrosive or chemically aggressive by-products (e.g., halogen-containing species or organic ligands) may be generated, which can complicate reactor design and impact long-term coating stability if not fully removed during processing. Despite these limitations, ALD remains a highly attractive approach for engineering highly controlled, conformal antibacterial coatings on titanium-based biomaterials, as demonstrated in multiple studies [26,27,106,113,115].

#### 6.1.4. Summary of Surface Modification

The rapid development of biomaterials focuses not only on the development of new alloys but, above all, on advanced methods of modifying implant surfaces, which play a key role in improving their physicochemical, biological, and antibacterial properties. Among the most important advantages of these strategies is the ability to simultaneously promote osseointegration and reduce the risk of peri-implant infections by reducing bacterial adhesion and inhibiting biofilm formation. Depending on the biological, physical–mechanical, chemical, or composite method used, it is possible to achieve a surface that promotes bone cell adhesion, enhances bioactivity, and increases resistance to corrosion and wear. Particularly important are combined approaches and modern techniques, such as atomic layer deposition (ALD), which allow for precise control of the thickness, composition, and uniformity of coatings even on surfaces with complex geometries. Additionally, these methods allow for imparting antimicrobial properties to implants, including through the incorporation of silver nanoparticles or active metal oxides. Despite numerous advantages, implant surface modifications also come with certain drawbacks. In the case of physical and mechanical methods, there is a risk of excessively increasing surface roughness, which may weaken the strength of the implant–tissue bond. Some coatings, particularly those of greater thickness, may undergo delamination, leading to inflammatory reactions. Chemical methods, while effective in improving bioactivity, may require the use of aggressive reagents, which complicates the manufacturing process. In turn, advanced techniques such as ALD, despite their high precision, are costly and require specialized equipment. A significant limitation remains the dependence of modification effectiveness on clinical conditions and the specific application of the implant. There is no universal method that would simultaneously ensure optimal mechanical, biological, and antibacterial properties. Furthermore, the long-term stability of coatings, their wear resistance, and their impact on the release of metal ions still require further research. In particular, a better understanding of the interactions between the modified surface and the biological environment, including the body’s immune response, is necessary. In clinical practice, implant surface modifications are widely used in orthopedics and dentistry, where rapid and durable osseointegration and the minimization of infection risk are of critical importance. Contemporary approaches focus on designing bioactive and antibacterial surfaces that not only support the healing process but also actively counteract bacterial colonization. Particularly promising are technologies enabling the precise shaping of surface properties at the nanoscale, which opens up new possibilities in the design of next-generation implants with enhanced clinical efficacy.

## 7. Conclusions

Previous studies on antibacterial coatings on titanium implants have focused primarily on the use of well-known metals such as silver, copper, and zinc, as well as organic compounds and hybrid materials. Although these approaches demonstrate effective antimicrobial activity, their application is often associated with the risk of cytotoxicity or limited coating durability. Tin dioxide (SnO_2_) is gaining increasing attention as a coating material. It is characterized by strong bactericidal and bacteriostatic properties while maintaining low toxicity toward host cells. In addition, the tin dioxide structure enables the formation of surfaces with anti-adhesive properties, effectively limiting bacterial biofilm formation. For this reason, an effective solution appears to be the combination of tin (Sn), exhibiting bactericidal properties, with the atomic layer deposition (ALD) of thin films, enabling precise, layer-by-layer deposition of nanometer-thick coatings on implant surfaces, even those with complex and porous structures. Due to sequential deposition involving the use of highly reactive precursors and eliminating the risk of uncontrolled layer growth, the ALD technology is distinguished by providing uniform coverage of the entire surface, even in hard-to-reach areas. Studies confirm that these coatings reduce metal ion release and surface wear of implants, which may limit inflammatory responses. As a result, ALD represents an effective surface modification method providing appropriate properties and antibacterial performance.

## Figures and Tables

**Table 1:** Types of infection and etiological agents associated with biofilm formation on biomaterial surfaces [54].

Type of Infection	Etiological Agent
Surgical site infections (SSIs)	*Staphylococcus aureus*, *Staphylococcus epidermidis*, *Staphylococcus saprophyticus*, *Escherichia coli*
Infections associated with dental implants	*Staphylococcus aureus*, *Staphylococcus epidermidis*, *Streptococcus* spp., *Escherichia coli*
Infections associated with orthopedic implants	*Staphylococcus aureus*, *Staphylococcus epidermidis*, *Cutibacterium acnes*, *Escherichia coli*
Infections associated with urinary catheters	*Pseudomonas aeruginosa*, *Staphylococcus saprophyticus, Escherichia coli*
Infections associated with artificial heart valves	*Staphylococcus epidermidis*, *Staphylococcus aureus*, *Enterococci*, *Escherichia coli.*
Infections associated with vascular grafts and catheters	*Staphylococcus epidermidis*, *Staphylococcus aureus*, *Enterobacteriaceae*, *Escherichia coli*

**Table 2:** Chemical composition (wt%) of selected titanium alloys according to the requirements of ISO 5832-3 [77], ISO 5832-11 [78], and ASTM F1713-08 [79].

Titanium Alloy	% Mass Elements									
	C	H	O	N	Fe	Nb	Al.	V	Zr	Ti
Ti-6Al-4V	≤0.08	≤0.015	≤0.2	≤0.05	≤0.3	-	5.5–6.75	3.5–4.5	-	balance
Ti-6Al-7Nb	≤0.08	≤0.009	≤0.2	≤0.05	≤0.25	6.5–7.5	5.5–6.75	-	-	balance
Ti-13Nb-13Zr	≤0.08	≤0.015	≤0.016	≤0.05	≤0.25	12.5–14.0	-	-	12.5–14.0	balance

**Table 3:** Mechanical properties of selected titanium alloys according to the requirements of ISO 5832-3 [77], ISO 5832-11 [78], and ASTM F1713-08 [79].

Titanium Alloy	R_m_ [MPa]	R_e_ [MPa]	A [%]	Z [%]	E [GPa]
Ti–6Al–4V	860	780	10	-	114
Ti–6Al–7Nb	900	800	10	25	100–110
Ti–13Nb–13Zr	973–1037	836–908	10–16	27–53	79–84

## Data Availability

No new data were created or analyzed in this study. Data sharing is not applicable to this article.

## Abbreviations

The following abbreviations are used in this manuscript:

AEW	acidic electrolyzed water
AFM	atomic force microscopy
ALD	atomic layer deposition
CVD	chemical vapor deposition
cp-Ti	commercially pure titanium
DNase I	deoxyribonuclease I
EPD	electrophoretic deposition
EPS	extracellular polymeric substances
GO	graphene oxide
HA	hydroxyapatite
MAO	micro-arc oxidation
PCL	polycaprolactone
QS	quorum sensing
SAEW	slightly acidic electrolyzed water
SEM	scanning electron microscopy
Si	silicon
XPS	X-ray photoelectron spectroscopy
XRD	X-ray diffraction
